# Intergenerational Association of Short Maternal Stature with Stunting in Yanomami Indigenous Children from the Brazilian Amazon

**DOI:** 10.3390/ijerph18179130

**Published:** 2021-08-30

**Authors:** Jesem Douglas Yamall Orellana, Giovanna Gatica-Domínguez, Juliana dos Santos Vaz, Paulo Augusto Ribeiro Neves, Ana Claudia Santiago de Vasconcellos, Sandra de Souza Hacon, Paulo Cesar Basta

**Affiliations:** 1 Leônidas e Maria Deane Institute, Oswaldo Cruz Foundation, Rua Teresina, 476, Adrianópolis, Manaus 69057-070, Brazil; jesem.orellana@gmail.com; 2 Postgraduate Program in Epidemiology, Faculty of Medicine, Federal University of Pelotas, Rua Marechal Deodoro, 1160-3° Piso, Centro, Pelotas 96020-220, Brazil; giovagatica@gmail.com (G.G.-D.); juliana.vaz@gmail.com (J.d.S.V.); paugustorn@gmail.com (P.A.R.N.); 3 Faculty of Nutrition, Federal University of Pelotas, Rua Gomes Carneiro, 1, Centro, Pelotas 96010-610, Brazil; 4 Laboratory of Professional Education in Health Surveillance, Joaquim Venâncio Polytechnic School of Health, Oswaldo Cruz Foundation, Av. Brasil, 4365-Manguinhos, Rio de Janeiro 21040-900, Brazil; anacsvasconcellos@gmail.com; 5 Samuel Pessoa Department of Endemics, National School of Public Health, Oswaldo Cruz Foundation, Rio de Janeiro 21041-210, Brazil; sandrahacon@gmail.com

**Keywords:** poverty areas, undernutrition, indigenous populations, intergenerational relations, epidemiologic determinants

## Abstract

To describe the factors associated to stunting in <5-year-old Yanomami Brazilian children, and to evaluate the association of short maternal stature to their offspring’s stunting. A cross-sectional study carried out in three villages in the Yanomami territory. We performed a census, in which all households with children < 5-years-old were included. The length/height-for-age z-score <−2 standard deviations was used to classify the children as stunted. Short maternal height was defined as <145 cm for adult women, and <−2 standard deviations of the height-for-age z-score for adolescent women. We used adjusted Poisson regression models to estimate prevalence ratios (PR) along the 90% confidence interval. We evaluated 298 children. 81.2% of children suffered from stunting and 71.9% of the mothers from short stature. In the bivariate analysis, a significant association of stunting with short maternal stature, gestational malaria and child’s place of birth were observed. Considering the variables of the children under five years of age, there were significant associations with age group, the child’s caregiver, history of malaria, pneumonia, and malnutrition treatment. In the adjusted hierarchical model, stunting was 1.22 times greater in the offspring of women with a short stature (90% CI: 1.07–1.38) compared to their counterparts. Brazilian Amazonian indigenous children living in a remote area displayed an alarming prevalence of stunting, and this was associated with short maternal height, reinforcing the hypothesis of intergenerational chronic malnutrition transmission in this population. In addition, children above 24 months of age, who were born in the village healthcare units and who had had previous treatment in the past for stunting presented higher rates of stunting in this study.

## 1. Introduction

Stunting, defined as the height-for-age deficit, not only raises the risk of mortality and incapacity in childhood [1], but is also associated with short stature in adulthood, neurocognitive development impairment, and long-term reduction in human capital [2,3,4]. The 2030 Agenda for sustainable development aims to eliminate all forms of malnutrition in children under 5 years of age (Goal 2.2) and reduce inequalities, ensuring no one is left behind (Goal 10) [5]. Unfortunately, it is expected that the Covid-19 pandemic will not only affect the discreet advancements towards eliminating all forms of malnutrition in children under 5 years of age, but it will also widen already existing socioeconomic inequalities [6,7].

In general, the indigenous peoples of Latin America have been an ethnical group disproportionately less favored [8]. Indigenous children present high prevalence of stunting when compared to non-indigenous children [9,10]. In Brazil, it is estimated that stunting affects approximately 6% of children under 5 years of age [11], with important regional inequalities, since stunting prevalence reaches 8.5% and 3.9% in non-indigenous children living in the North and South regions, respectively [11]. In indigenous people the situation is even worst, as stunting occurs in approximately 26% of indigenous Brazilian children, especially in the North region (nearly 41% of children experiencing stunted growth) [12]. The comparative analysis reveals deep inequalities between Brazilian macro-regions and different ethnical groups in the country.

There is evidence that short maternal height negatively influences the growth of linear offspring [13], which persists through adulthood [13,14,15]. Recent studies showed that growth-faltering begins during pregnancy [14]. Therefore, the need for measures to intervene and prevent the intergenerational effects (e.g., epigenetics, metabolic programming due to alterations, amongst others) associated with short maternal stature is crucial, especially during the “first 1000 days window”. The interventions must seek to break the poverty cycle and the consequential intergenerational deficits in adult human capital [14].

Recognizing the worrisome nutritional situation in indigenous children, the investigation of its determinants remains an important challenge not only for sanitary authorities, but also for healthcare workers and professionals [16]. Therefore, the objective of this study was to describe the factors associated with stunting in <5-year-old Yanomami Brazilian children, and to evaluate the association of short maternal stature to their offspring’s stunting.

## 2. Materials and Methods

### 2.1. Study Area and Population

**Figure 1:**
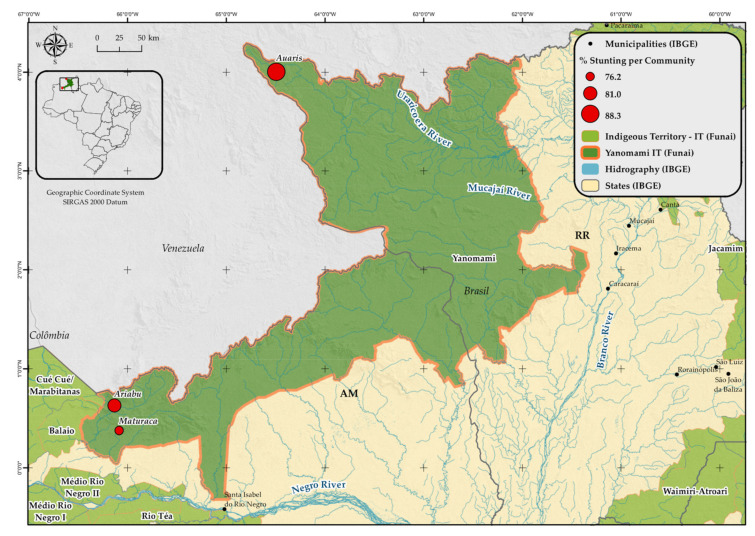
Studied villages in the Yanomami Indigenous Territory (YIT), according to the prevalence of stunting. Roraima and Amazonas states, Brazilian Amazon, 2018–2019.

In Brazil, the Yanomami population is comprised of nearly 28,000 individuals, distributed over 360 villages. The Yanomami Indigenous Territory (YIT) covers 9,664,975 hectares, bordering with Venezuela. This study was conducted with children under 5 years of age and their mothers, in the administrative regions *Auaris* and *Maturacá* in the Brazilian Amazon. Two villages in the *Maturacá* region were included in the study, namely *Ariabú* and *Maturacá* (Figure 1).

The *Auaris* region is located in the extreme north of the state of Roraima, one of the areas with the highest population density of the YIT (Figure 1). It is exclusively accessed by air from the state capital, *Boa Vista*. In turn, *Maturacá* is based in the Amazonas state and can be accessed by air from *Boa Vista* or *São Gabriel da Cachoeira,* Amazonas, or by land and boat from *São Gabriel da Cachoeira*.

### 2.2. Study Design

A cross-sectional census study was conducted between 9 and 22 December 2018 in the *Auaris* region and between 6 and 27 February 2019 in *Ariabú* and *Maturacá* villages. For this study, semi-structured interviews were conducted by key-informants (women/mothers or people responsible for the household) using data collection questionnaires. When key-informants did not speak Portuguese, a local translator mediated the interviews. The consent to participate was obtained through signature (or fingerprint) of the informed consent.

### 2.3. Maternal and Child Anthropometry

To measure the height and weight of participants we used the following equipment: vertical anthropometer or Alturexata^®^ stadiometer with an infantometer adaptor with a precision of 0.1 cm (length measure) and a Seca^®^ portable digital scale (model 877) with maximum capacity of 150 kg and a precision of 0.1 kg.

We used the WHO-Anthro^®^ [17] and WHO-Anthro Plus^®^ [18] programs to build the anthropometric length/height-for-age index in z-scores of children under 5 years of age and adolescent women, respectively.

We classified children as stunted and adolescent women with a short stature if they presented a length/height-for-age z-score less than −2 standard deviations of the median population of reference, according to the WHO Growth Charts.

Anthropometric information of six children was excluded from the analysis due to congenital malformations and implausible values of the length/height-for-age z-scores. No adolescent mother showed implausible z-score values [17,18]. For women older than 18 years of age, we considered a height of less than 145 cm as constituting a short stature [19].

### 2.4. Sociodemographic, Maternal and Child Characteristics

The following sociodemographic characteristics were considered: (i) region/village of residence (*Auaris; Maturacá; Ariabú*); (ii) parent knowledge (read or write) in Portuguese (yes; no); (iii) home source of income (Federal, Statal/Municipal Governments, Others or No Income); (iv) household water source for human consumption (well; river/stream); (v) waste management (thrown in the forest/river; burnt/buried); (vi) household wall-type (wood/brick; clay; straw/no wall); (vii) household density (1 to 6; 7 to 9; ≥10 people).

The maternal characteristics considered were: (i) age group (13 to 24; 25 years or older); (ii) short stature (yes; no); (iii) number of antenatal consultations (0 to 3; 4 to 6; 7 to 9); (iv) gestational malaria history (yes; no); (v) children’s place of birth (house/forest; village’s healthcare units; *Casa de Saúde do Índio*—CASAI/Hospital).

With respect to the children, we considered the following characteristics: (i) sex (male; female); (ii) age group (≤23; 24 to 59 months); (iii) child’s main caregiver (mother or father, other family member); (iv) weight at birth (<2500 g; ≥2500 g); (v) previous pneumonia treatment (yes; no); (vi) previous malnutrition treatment (yes; no); (vii) previous malaria treatment (yes; no).

### 2.5. Statistical Analysis

Differences between proportion were assessed using the Pearson chi-square test. The Crude-Poisson regression, adjusted to covariables with consistent covariance matrix estimator type HC2, was used to estimate the Prevalence Ratio (PR) for stunting [20].

In the crude analysis, we selected variables with a *p* value < 0.20 to further adjust the analysis, using an hierarchical framework based on previous studies [21]. The conceptual hierarchical framework, established a priori, was structured into four levels and the introduction of variables in the models obeyed a hierarchical sequence. The hierarchical levels included were: 1st level (sociodemographic characteristics): region/village of residence and type of wall; 2nd level (maternal characteristics): gestational malaria and child’s place of birth; 3rd level (child characteristics): age group and child main caregiver; 4th level (health history of the child): child’s previous malaria treatment.

After the first selection, we used the backward method to select variables associated at *p*-values < 0.10 in each level to the final adjust model. Pre-selected variables in each level were retained in the subsequent models, independent of their *p*-value. Additionally, the interaction between household wall-type and place of birth was tested in the final adjusted model, considering a *p*-value < 0.10. 

The villages included in the present study were based in hard-to-reach areas of the Brazilian Amazon. Due to the small sample size (*n* = 298), we adopted *p*-values of 0.10 and 0.20 as statistically significant in order to guarantee that any effects from the studied variables could be tested. However, it is worth remembering that we carried out a census in the investigated villages, and all children under 5 years of age were included in the study. 

Data analysis was performed using the statistical software R, version 3.3.2 [22].

## 3. Results

The prevalence of stunting among indigenous children under 5 years of age was 81.2% (Table 1), varying greatly across villages, with the highest burden in *Auaris* (88.3%) and the lowest in *Ariabú* (76.2%). Considering only children older than 12 months of age, stunting prevalence was superior to 85%, and in the age-group ranging from 24 to 59 months of age stunting was superior to 90%, regardless of region/village. The prevalence of short maternal stature was 71.9% (mean maternal height of 143.0 cm; standard deviation = 4.3) (Table 2). Similarly to stunting in children, the highest burden of short maternal height was seen in *Auaris* (87.7%) and the lowest in mothers in *Ariabú* (65.0%) regions/villages.

When comparing the sociodemographic characteristics between the villages, we observed that most fathers in *Auaris* did not know how to read and write in Portuguese (44.9%). Similarly, the stunting frequencies were higher among families that dispose household waste in the river or the forest (92.4%), households with no regular income (65.4%), and households with clay walls (74.4%) (Table 2). Furthermore, regarding maternal and perinatal variables, only 1.7% of mothers in *Auaris* attended between 7 and 9 antenatal visits, and only 6.4% of mothers in *Auaris* gave birth in a healthcare facility (hospital/CASAI).

Regarding sociodemographic variables, only household wall-type was shown to be significantly associated with stunting (Table 3). We found a significant association between stunting with short maternal stature, gestational malaria, and the child’s place of birth. Lastly, for variables related to child health, stunting was associated with age, the child’s caregiver, previous malaria, pneumonia, and previous undernutrition treatment.

In the hierarchical multiple regression analysis, which included the interactive term between household wall-type and child’s place of birth, the final model indicated a stunting risk of 22% (7–38%) higher in children whose mothers with a short stature, compared to those with normal stature (Table 4).

## 4. Discussion

We described an alarming prevalence of stunting among Brazilian indigenous children living in the Amazon. Additionally, we found a significant association between short maternal stature and stunting in the offspring. Such findings reinforce the hypothesis of intergenerational transmission of stunting in Yanomami indigenous people, highlighting the precarity of health and nutrition conditions in these settings.

Stunting prevalence varies markedly across regions of the world. Recent evidence suggests that stunting occurs in 39.0% and 55.0% of African and Asian children under five, respectively [6,23]. In Brazil, a nationwide survey showed that 40.8% of indigenous children under five years of age, living in the North region, suffered from stunting [12]. In contrast, stunting prevalence of 6.3% and 5.7% were reported in the same period for non-indigenous boys and girls under 5 years of age in the same area, respectively [11], revealing huge inequalities in the nutritional status of indigenous children in Brazil. Despite the chronic patterns of stunting among indigenous people globally, a prevalence of stunting above 80% have only been reported among Yanomami children, highlighting the critical nutritional status of this group [24,25].

Scientific research on the determinants of the nutritional status in indigenous children under five years of age can improve public policies and reduce social inequalities in health, fostering social protection mechanisms. At the same time, the association between short maternal stature and stunting in our study showed not only an intergenerational effect but reinforces previous findings of the high burden of stunting in Yanomami children, as well as the need for interventions aimed at breaking down the cycle of sickness and death that remain for decades [24,25,26,27,28,29,30]. 

The critical scenario revealed in this study confirms that the high rates of stunting persist for a long time in the Yanomami Indigenous territory as a result of not only a permanent state of food insecurity but also due to structural racism historically imposed by the Government. In contrast with the goals of the 2030 Agenda for sustainable development [5], which aim to eliminate all forms of malnutrition in children under five years of age and reduce inequalities, leaving no one behind, and the United Nations Declaration on the Rights of Indigenous Peoples (UNDRIP) [31,32], the Brazilian Government has not fulfilled its duty. 

Throughout history, the indigenous people have been marginalized, discriminated against, and exploited by Western society. In turn, the Government has not implemented inclusive and sustainable public policies to reduce poverty and starvation and promote reparation for the violations suffered by these peoples. In addition, the Government continues to deny them access to essential public services, such as health, sanitation, education, employment, and income, as well as the rights to self-determination and the control over natural resources existing on their traditional territories. Moreover, in the last couple of years, several bills have been presented to the parliament in order to reduce rights assured in the Brazilian Federal Constitution. 

Therefore, we consider that the path to sovereignty should be built in another way, putting on the indigenous people at the center of the debate, valorizing their culture and their traditional knowledge, in order to attain a fairer and equitable society, where no child, no citizen and no nations will be left behind.

A recent analysis regarding ethnic inequalities in the prevalence of stunting in children under 5 years of age in 13 Latin American countries showed a highly unfavorable situation for indigenous populations. The polled prevalence ratio of stunting for all countries was 1.34 (CI 95% 1.28 to 1.39) for indigenous children compared to non-indigenous children, even after adjustments were made to household wealth and place of residence [9]. Furthermore, another study conducted in Latin American countries showed a low coverage of healthcare services for indigenous women and children when compared to non-indigenous people, independent of wealth or place of residence [33]. Although these studies did not capture the specific space-temporal contextual particularities, they reinforce social inequality and precarious access to healthcare and sanitary services by indigenous people, even though such items are considered part of universal rights [32,33]. In Brazil, indigenous people are a minority, and they represent less than 0.5% of the country’s population, which makes the adoption and implementation of a broad intercultural healthcare model crucial [34].

The well-known intergenerational transmission of poverty and some critical prenatal determinants play a significant role in the high burden of stunting in many parts of the world. The more precarious the living conditions, the worse the malnutrition indicators in children, notably the stunting cases [35]. This phenomenon is consistent with the social determinants of the child malnutrition model developed by UNICEF [36], that states that the precarious conditions of health and maternal diet, before conception and during pregnancy, as well as a persistent exposure to a poor diet, considering quantity and quality of the food (i.e., food with low energy, protein, and micronutrients content), in addition, recurrent infections of infancy are each among the leading causes of stunting. On the other hand, other causes of stunting include food insecurity, inadequate feeding practices, an unhealthy domestic environment, inadequate healthcare service access, and poverty [36].

Food insecurity leads to the reduction in quantity and quality of consumed foods, as well as changing eating habits, due to lack of money or the absence of other material resources. This combination of factors acting together negatively affects the nutritional status, health and welfare of families [36]. There is evidence that indigenous children between 6 and 23 months of age in Latin American countries receive maternal milk for a longer period of time, however, the complementary foods available for these children are low in quality. Therefore, the availability of maternal milk for a longer period of time does not seem sufficient to meet the nutritional needs of the children in this age group. Consequently, these children are more susceptible to catch-up delays in growth [9].

It is important to point out that, historically, indigenous people have been exposed to high loads of infectious and parasitic diseases, both in terms of frequency and seriousness of the reported clinical conditions [37,38]. In this sense, parasitic infestation, gastrointestinal infections, and enteric environmental disfunctions are especially important due to negative effects in nutrient digestion and absorption, threatening not only the growth potential, but also raising the risk of death in indigenous children [39,40,41]. The precarious sanitary conditions in the indigenous villages in Latin America [42], as well as those observed in the studied Yanomami children—including the limited access to potable drinking water and inadequate household waste destination—help to understand not only the elevated and permanent exposure of these children to infectious and parasitic diseases [30], but the severity of the nutritional situation. The precarious nutritional status of the Yanomami children living in Brazil and Venezuela [24,25,26,27,28,29] has been systematically denounced for at least three decades.

One of the long-term consequences of stunting in childhood is the short stature in adulthood [43]. Mothers with a short stature in adulthood can give birth to tiny babies due to an insufficient supply of nutrients or due to an unfavorable intrauterine environment—due to lack of food—for adequate fetal growth [44]. Our findings reinforce another record from Orellana et al. [24] that shows an association between short maternal stature and severe stunting in Yanomami children under five years of age who reside in other regions of the YIT in Brazil. In the same study, the authors also revealed a stunting prevalence of 83.8%, and that children of mothers with height < 145 cm presented higher prevalence of stunting (PR: 2.1; CI 95%: 1.2–3.6) compared to children of mothers with stature ≥145 cm. 

In turn, a study using data from birth cohorts carried out in five low- or middle-income countries (Brazil, Guatemala, India, Philippines, and South Africa) showed that mothers with a stature shorter than 150.1 cm presented a 3.2 times higher probability of having children with stunting at 2 years of age (CI 95%: 2.8–3.6) and 4.7 times higher in the adulthood (CI 95%: 4.1–5.4), when compared to mothers with a higher stature [13].

Other potential threats help explain the precarious nutritional conditions of the Yanomami Brazilian children, such as the invasions of the traditional territories by illegal miners, loggers, grabbing, and other criminals searching of wealth in the Amazon Forest [45,46]. All factors previously mentioned can simultaneously increase the risks of food insecurity and the maintenance of stunting in children under five years of age and all family members living in the region.

Despite the illustrative findings of this investigation, consistent with the specialized literature, it is important to consider the limitations. It was not possible to evaluate children who were receiving healthcare treatments outside of the villages at the time of our visits. On the one hand, this could have contributed to underestimation of stunting prevalence in our study. On the other hand, the reduced number of villages included in this study does not represent the nutritional status of all the children who live in the YIT, thus requiring caution in the generalizations of the results. Although all of the house visits were conducted with a local translator, the information bias cannot be disregarded, as well as memory bias, especially in questions related to healthcare services access and former sickness in older children.

Aside the aforementioned limitations, it is worth highlighting that although the number of Yanomami children evaluated (*n* = 298) could be considered small, it was expressive since these children live in regions with difficult access and that, in certain circumstances, these children can pass months without regular visits by healthcare teams. Under the methodological point of view, the exposure and the outcome evaluated in this study were measured following quality recommendations standards by WHO. Despite the cross-sectional nature of our data, we can say that the reverse causality phenomenon did not affect our association estimates, since the short maternal stature exposure was already defined before pregnancy and childbirth, which eliminates the exposure-outcome temporality dilemma in our data. Finally, even without excluding the possibility of residual confusion, we adopted robust data analysis methods by a hierarchical approach, using a measure of appropriate association to sectional studies.

## 5. Conclusions

The high prevalence of stunting recorded in children under 5 years of age in the Yanomami Indigenous territory, especially those living in the *Auaris* region, reveals an alarming and unprecedented situation of health neglect. Therefore, it is essential to remember that Article 21 of the United Nations Declaration on the Rights of Indigenous Peoples assures that: “Indigenous peoples have the right, without discrimination, to the improvement of their economic and social conditions, including, among other things, in the areas of education, employment, vocational training and retraining, housing, sanitation, health and social security” [33].

In conclusion, even if the birth of many generations is necessary to eliminate the effects of intergenerational short stature, it is vital to understand that specific, broad, and inclusive nutritional interventions and programs are essential to prevent stunting in an ethnic and culturally differentiated context, especially if they are addressed in the first 1000 days of a child’s life. This period is considered a unique window of opportunity, especially in indigenous children, seriously affected by stunting. Therefore, effective multisectoral measures must be implemented to eradicate stunting in the Yanomami Territory.

Finally, we consider it equally essential to create sustainable projects by the Federal Government in close collaboration with non-governmental organizations and indigenous associations. These projects must propose strategies to assure regular income generation with the participation of local society in managing the resources, assuring self-identification patterns, cultural valorizing, respect to ancient knowledge, and traditional foods. The ultimate goal should be to reach food sovereignty and social inclusion, guaranteeing basic water supply services, sanitation in villages, and regular access to health services.

## Figures and Tables

**Table 1:** Stunting characterization in children under 5 years of age, according to the villages of residence. Yanomami Indigenous Territory, Brazilian Amazon, 2018–2019.

	Stunting											
Age Group (Months)	*Auaris*			*Maturacá*			*Ariabú*			Total		
	*N*	*n*	%	*N*	*n*	%	*N*	*n*	%	*N*	*n*	%
≤11	12	7	58.3	24	9	37.5	25	7	28.0	61	23	37.7
12 to 23	16	16	100.0	18	16	88.9	20	17	85.0	54	49	90.7
24 to 35	12	12	100.0	22	22	100.0	17	17	100.0	51	51	100.0
36 to 59	37	33	89.2	52	47	90.4	43	39	90.7	132	119	90.2
Total	77	68	88.3	116	94	81.0	105	80	76.2	298	242	81.2

*N*—total number of children in the evaluated age group; *n*—number of children with stunting.

**Table 2:** Sociodemographic, maternal and children under five years of age characteristics, according to the village of residence. Yanomami Indigenous Territory, Brazilian Amazon, 2018–2019.

	Villages of Residence			
	*Ariabú*	*Auaris*	*Maturacá*	*p*-Value *
	*n* (%)	*n* (%)	*n* (%)	
Sociodemographic variables				
Read or write in Portuguese				<0.001
No	11 (10.7%)	35 (44.9%)	16 (14.0%)	
Yes	92 (89.3%)	43 (55.1%)	98 (86.0%)	
Source of Income				<0.001
Federal	12 (11.4%)	18 (23.1%)	11 (9.3%)	
Municipal/Statal	24 (22.9%)	4 (5.1%)	25 (21.2%)	
Other	8 (7.6%)	5 (6.4%)	26 (22.0%)	
No income	61 (58.1%)	51 (65.4%)	56 (47.5%)	
Drinking water source				<0.001
River or stream	82 (78.1%)	55 (70.5%)	17 (14.4%)	
Well	23 (21.9%)	23 (29.5%)	101 (86.6%)	
Household waste destination				<0.001
Burnt or buried	38 (36.2%)	6 (7.6%)	90 (76.9%)	
Forest or river	67 (63.8%)	73 (92.4%)	27 (23.1%)	
Household wall type				<0.001
Wood/brick	40 (38.1%)	7 (9.0%)	46 (49.5%)	
Clay	52 (49.5%)	58 (74.4%)	61 (35.7%)	
Straw/no wall	13 (12.4%)	13 (16.6%)	09 (25.7%)	
Household density (number of people)				0.035
1 to 6	32 (30.5%)	18 (23.7%)	40 (33.9%)	
7 to 9	27 (25.7%)	27 (35.5%)	47 (39.8%)	
10 or more	46 (43.8%)	31 (40.8%)	31 (26.3%)	
Maternal and perinatal variables				
Age group (years)				0.118
13.0 to 24.9	42 (40.0%)	20 (27.7%)	49 (41.5%)	
25.0 or more	63 (60.0%)	53 (72.6%)	69 (58.5%)	
Short maternal stature				0.002
No	35 (35.0%)	9 (12.3%)	37 (32.2%)	
Yes	65 (65.0%)	64 (87.7%)	78 (67.8%)	
Number of antenatal consultations				<0.001
0 to 3	19 (21.1%)	31 (52.5%)	22 (20.2%)	
4 to 6	50 (55.6%)	27 (45.8%)	71 (65.1%)	
7 to 9	21 (23.3%)	1 (1.7%)	16 (14.7%)	
Gestational malaria				0.205
No	89 (84.8%)	68 (93.2%)	100 (85.5%)	
Yes	16 (15.2%)	5 (6.8%)	17 (14.5%)	
Place of birth				<0.001
Household/Forest	57 (53.8%)	71 (91.0%)	68 (59.6%)	
Primary Healthcare Center (villages)	27 (25.5%)	2 (2.6%)	23 (20.2%)	
Hospital/CASAI ^1^	22 (20.8%)	5 (6.4%)	23 (20.2%)	
Child’s variables				
Sex				<0.001
Female	69 (65.7%)	26 (32.9%)	81 (68.6%)	
Male	36 (34.3%)	53 (67.1%)	37 (31.4%)	
Age group (months)				0.534
≤23	46 (43.4%)	29 (36.2%)	44 (37.3%)	
24 to 59	60 (56.6%)	51 (63.8%)	74 (62.7%)	
Child’s caregiver				<0.001
Mother or father	80 (75.5%)	40 (50.6%)	101 (87.8%)	
Another family member	26 (24.5%)	39 (49.4%)	14 (12.2%)	
Low weight at birth				0.977
No	93 (89.4%)	61 (88.4%)	103 (88.8%)	
Yes	11 (10.6%)	8 (11.6%)	13 (11.2%)	
Pneumonia treatment				0.069
No	70 (66.7%)	39 (50.0%)	66 (56.9%)	
Yes	35 (33.3%)	39 (50.0%)	50 (43.1%)	
Undernutrition treatment				0.127
No	93 (87.7%)	60 (76.9%)	94 (79.7%)	
Yes	13 (12.3%)	18 (23.1%)	24 (20.3%)	
Malaria treatment				0.239
No	97 (91.5%)	67 (84.8%)	108 (91.5%)	
Yes	9 (8.5%)	12 (15.2%)	10 (8.5%)	
Stunting prevalence				0.118
No	25 (23.8%)	9 (11.7%)	22 (19.0%)	
Yes	80 (76.2%)	68 (88.3%)	94 (81.0%)	

*n* = number of cases observed in each variable, according to the village of residence; * *p*-value refers to Pearson’s chi-square test; ^1^ CASAI (*Casa de Saúde do Índio*): Unit of support to the indigenous people who live in villages during healthcare attendance in urban centers.

**Table 3:** Proportion of children under five years of age with and without stunting according to sociodemographic, maternal and child variables. Yanomami Indigenous Territory, Brazilian Amazon, 2018–2019.

	Stunting		
	No	Yes	*p*-Value *
	*n* (%)	*n* (%)	
Sociodemographic variables			
Parent knows how to read or write in Portuguese			0.877
No	11 (20.0%)	49 (20.9%)	
Yes	44 (80.0%)	185 (79.1%)	
Source of Income			0.305
Federal	11 (19.6%)	30 (12.6%)	
Municipal/Statal	12 (21.4%)	41 (17.2%)	
Other	8 (14.3%)	30 (12.6%)	
No income	25 (44.6%)	138 (57.7%)	
Drinking water source			0.799
River or stream	28 (50.0%)	124 (51.9%)	
Well	28 (50.0%)	115 (48.1%)	
Household waste destination			0.409
Burnt or buried	28 (50.0%)	105 (43.9%)	
Forest or river	28 (50.0%)	134 (56.1%)	
Household wall type			0.063
Wood/brick	25 (44.6%)	68 (28.7%)	
Clay	27 (48.2%)	141 (59.5%)	
Straw/no wall	4 (7.1%)	28 (11.8%)	
Household density (number of people)			0.770
1 to 6	17 (30.4%)	71 (30.0%)	
7 to 9	17 (30.4%)	83 (35.0%)	
10 or more	22 (39.3%)	83 (35.0%)	
Maternal and perinatal variables			
Age group (years)			0.390
13.0 to 24.9	17 (32.1%)	91 (38.4%)	
25.0 or more	36 (67.9%)	146 (61.6%)	
Short maternal stature			<0.001
No	28 (52.8%)	53 (23.1%)	
Yes	25 (47.2%)	176 (76.9%)	
Number of antenatal consultations			0.797
0 to 3	16 (31.4%)	54 (26.7%)	
4 to 6	28 (54.9%)	117 (57.9%)	
7 to 9	7 (13.7%)	31 (15.3%)	
Gestational malaria			0.071
No	52 (94.5%)	201 (85.5%)	
Yes	3 (5.5%)	34 (14.5%)	
Place of birth			0.027
House/Forest	33 (62.3%)	158 (66.1%)	
Primary Healthcare Center (villages)	5 (9.4%)	46 (19.2%)	
Hospital/CASAI ^1^	15 (28.3%)	35 (14.6%)	
Child variables			
Sex			0.479
Female	30 (54.5%)	144 (59.8%)	
Male	25 (45.5%)	97 (40.2%)	
Age group (months)			<0.001
≤23	43 (76.8%)	72 (29.8%)	
24 to 59	13 (23.2%)	170 (70.2%)	
Child’s caregiver			0.089
Mother or father	45 (83.3%)	170 (70.8%)	
Another family member	9 (16.7%)	70 (29.2%)	
Low weight at birth			0.779
No	46 (90.2%)	207 (88.8%)	
Yes	5 (9.8%)	26 (11.2%)	
Pneumonia treatment			<0.001
No	43 (78.2%)	126 (52.9%)	
Yes	12 (21.8%)	112 (47.1%)	
Undernutrition treatment			0.002
No	54 (96.4%)	189 (78.8%)	
Yes	2 (3.6%)	51 (21.2%)	
Malaria treatment			0.072
No	54 (96.4%)	213 (88.4%)	
Yes	2 (3.6%)	28 (11.6%)	

*n* = number of cases observed; * *p*-value refers to Poisson regression; ^1^ *Casa de Saúde do Índio*: Unit of support to the indigenous people who live in villages during healthcare attendance in urban centers.

**Table 4:** Crude and adjusted hierarchical models of the association between stunting in Yanomami children under five years old and short maternal height. Yanomami Indigenous Territory, Brazilian Amazon, 2018–2019.

Level	Variable	Crude PR (CI 80%)	*p*-Value	Adjusted PR (CI 90%)	*p*-Value
	Region/village of residence				
	*Ariabú*	1		1	
	*Auaris*	1.07 (1.06–1.27)	0.032	1.09 (0.97–1.22)	0.222
	*Maturacá*	1.07 (0.97–1.16)	0.385	1.04 (0.93–1.17)	0.563
1	Household wall type				
	Wood/brick	1		1	
	Clay	1.15 (1.05–1.26)	0.055	1.13 (1.01–1.26)	0.088
	Straw/no wall	1.20 (1.06–1.35)	0.053	1.19 (1.01–1.40)	0.082
	Gestational malaria				
	No	1		1	
	Yes	1.06 (1.07–1.25)	0.014	1.12 (1.01–1.25)	0.070 ^a^
2	Place of birth				
	Hospital/Casai	1		1	
	Forest	1.10 (1.04–1.34)	0.092	1.17 (0.99–1.38)	0.118 ^b^
	Primary Healthcare Center (in the villages)	1.11 (1.13–1.47)	0.015	1.26 (1.05–1.51)	0.041 ^b^
	Age group (months)				
	≤23	1		1	
	24 to 59	1.08 (1.35–1.63)	0.001	1.48 (1.30–1.67)	0.001 ^c^
3	Child’s caregiver				
	Mother or father	1		1	
	Other family member	1.06 (1.05–1.20)	0.034	1.10 (1.01–1.20)	0.080 ^d^
4	Malaria treatment				
	No	1		1	
	Yes	1.06 (1.09–1.26)	0.007	1.05 (0.95–1.17)	0.412 ^e^
	Child stunting (outcome)				
	No	1		1	
	Yes	1.34 (1.20–1.49)	0.001	1.22 (1.07–1.38)	0.012 ^f^

PR = Prevalence Ratio; ^a^ Adjusted by wall-type. ^b^ Adjusted by wall-type and gestational malaria. ^c^ Adjusted by wall-type, gestational malaria and place of birth. ^d^ Adjusted by wall-type, gestational malaria, place of birth and age group. ^e^ Adjusted by wall-type, gestational malaria, place of birth, age group and child’s caregiver. ^f^ Adjusted by wall-type, gestational malaria, place of birth, age group and child’s caregiver to the interaction between wall-type and place of birth.

## Data Availability

Data sharing not applicable.
